# Reduced Innate Immune Response to a *Staphylococcus aureus* Small Colony Variant Compared to Its Wild-Type Parent Strain

**DOI:** 10.3389/fcimb.2016.00187

**Published:** 2016-12-26

**Authors:** Judy J. J. Ou, Amanda J. Drilling, Clare Cooksley, Ahmed Bassiouni, Stephen P. Kidd, Alkis J. Psaltis, Peter J. Wormald, Sarah Vreugde

**Affiliations:** ^1^Department of Surgery-Otorhinolaryngology Head and Neck Surgery, Basil Hetzel Institute, The University of AdelaideAdelaide, SA, Australia; ^2^School of Biological Sciences, Research Centre for Infectious Disease, The University of AdelaideAdelaide, SA, Australia

**Keywords:** *Staphylococcus aureus*, small colony variant, innate immune response, epithelial cells, tissue remodeling

## Abstract

**Background:** *Staphylococcus aureus (S. aureus)* small colony variants (SCVs) can survive within the host intracellular milieu and are associated with chronic relapsing infections. However, it is unknown whether host invasion rates and immune responses differ between SCVs and their wild-type counterparts. This study used a stable *S. aureus* SCV (WCH-SK2^SCV^) developed from a clinical isolate (WCH-SK2^WT^) in inflammation-relevant conditions. Intracellular infection rates as well as host immune responses to WCH-SK2^WT^ and WCH-SK2^SCV^ infections were investigated.

**Method:** NuLi-1 cells were infected with either WCH-SK2^WT^ or WCH-SK2^SCV^, and the intracellular infection rate was determined over time. mRNA expression of cells infected with each strain intra- and extra-cellularly was analyzed using a microfluidic qPCR array to generate an expression profile of thirty-nine genes involved in the host immune response.

**Results:** No difference was found in the intracellular infection rate between WCH-SK2^WT^ and WCH-SK2^SCV^. Whereas, extracellular infection induced a robust pro-inflammatory response, intracellular infection elicited a modest response. Intracellular WCH-SK2^WT^ infection induced mRNA expression of *TLR2*, pro-inflammatory cytokines (*IL1B, IL6*, and *IL12*) and tissue remodeling factors (*MMP9*). In contrast, intracellular WCH-SK2^SCV^ infection induced up regulation of only *TLR2*.

**Conclusions:** Whereas, host intracellular infection rates of WCH-SK2^SCV^ and WCH-SK2^WT^ were similar, WCH-SK2^SCV^ intracellular infection induced a less widespread up regulation of pro-inflammatory and tissue remodeling factors in comparison to intracellular WCH-SK2^WT^ infection. These findings support the current view that SCVs are able to evade host immune detection to allow their own survival.

## Introduction

*Staphylococcus aureus* (*S. aureus*) is a common pathogen known to have the ability to adapt to different host environments. Some *S. aureus* strains are capable of switching to an alternative Small Colony Variant (SCV) phenotype, allowing their survival under stressful conditions (Proctor et al., 2006; Bui et al., 2015b). *S. aureus* SCVs have been identified for many decades (Jensen, 1957), but only sparked more research interest in recent years when they were found to be associated with chronic recurrent infections in both humans (Schneider et al., 2008; Maduka-Ezeh et al., 2012; Wolter et al., 2013; Tande et al., 2014; Masoud-Landgraf et al., 2016) and animals (Atalla et al., 2008; Alkasir et al., 2013).

Generally, the model is that SCVs are auxotrophic to haemin (Kohler et al., 2003; Seggewiss et al., 2006; Von Eiff et al., 2006), menadione (Von Eiff et al., 2006) or thymidine (Chatterjee et al., 2007; Maduka-Ezeh et al., 2012) leading to defects in energy production and tricarboxylic acid metabolism and hence their slower growth and smaller colony size (Proctor et al., 2006). SCVs also show reduced hemolysis, loss of pigmentation, and increased tolerance of antibiotics (Proctor et al., 1994). These features make the detection and treatment of SCVs challenging in the clinical setting.

*S. aureus* has been found to have the ability to invade mammalian cells (Clement et al., 2005). Different reports show that upon invasion of mammalian cells, *S. aureus* can switch to a SCV phenotype and thereby be better adapted to the hostile intracellular environment than the parental, non-SCV cell type (henceforth referred to as wild type) (Tuchscherr et al., 2011; Tan et al., 2014). Once inside the cell, *S. aureus* SCVs have been shown to switch off their toxin production enabling them to survive in the host for prolonged periods of time without inducing an immune response (Tan et al., 2014; Ou et al., 2016). The switch from wild type to SCV is therefore strongly associated with improved survival within the host and defense against the antimicrobial processes; antibiotics and host immune cells (Tuchscherr et al., 2010).

SCVs' detection and identification are not only challenging in the clinical situation. Their accurate study in the laboratory setting is also problematic as clinical SCV isolates are often highly unstable and can rapidly revert to the wild type phenotype. Therefore, the majority of studies conducted on SCVs use laboratory-generated mutant *S. aureus* strains rather than naturally derived SCVs (Kohler et al., 2003; Seggewiss et al., 2006). In a recent study, Bui et al. simulated the inflammatory environment by adding different concentrations of organic chemicals relevant to the host immune response (i.e., methylglyoxal) together with low nutrients and bacterial growth rate for a prolonged timeframe (Bui et al., 2015a,b). After 50 days of continuous incubation in these conditions of one particular *S. aureus* clinical strain, WCH-SK2^WT^, the bacterial population included an increased number (80% of the colonies) of *stable* SCVs (Bui et al., 2015a). This provides a valuable resource of a clinical isolate in a parental form and then in an experimentally useable SCV state.

Epithelial cells lining mucosal layers such as sinonasal and intestinal mucosa form an important physical barrier between hosts and the outside world. This mucosal surface is part of the first line innate defense as this is the site where pathogens interface with hosts. Previous *in vitro* studies have shown that epithelial cells infected with wild type *S. aureus* had increased expression of cytokines/chemokines including interleukin (IL)6 (Damm et al., 2006; Sachse et al., 2010), CXCL8 (Damm et al., 2006; Kohanski and Lane, 2015) and tumor necrosis factor (TNF) (Li et al., 2009; Kohanski and Lane, 2015) and tissue remodeling factors such as matrix metalloproteinases (MMPs) (Homma et al., 2015). An *in vitro* study by Tuchscherr et al. showed increased expression of C-C motif chemokine ligand (CCL) 5, C-X-C motif ligand (CXCL) 10 and 11, and intracellular adhesion molecule (ICAM)1 when endothelial cells were infected with wild type *S. aureus* but not *S. aureus* SCVs (Tuchscherr et al., 2010). Whilst these studies invariably showed an induction of a host immune response upon *S. aureus* infection *in vitro*, there was no differentiation between the host immune response induced by extracellular vs. intracellular *S. aureus*. The aim of this study was to identify whether there is a difference in the intracellular infection rate of airway epithelial cells between WCH-SK2^WT^ and WCH-SK2^SCV^ as well as the cellular immune response that they induce 24 h after internalization.

## Materials and methods

### Ethics

Ethics approval for this study was granted by the Human Research Ethics Committee of The Queen Elizabeth Hospital (South Australia, Australia).

### Bacterial strains

**Figure 1:**
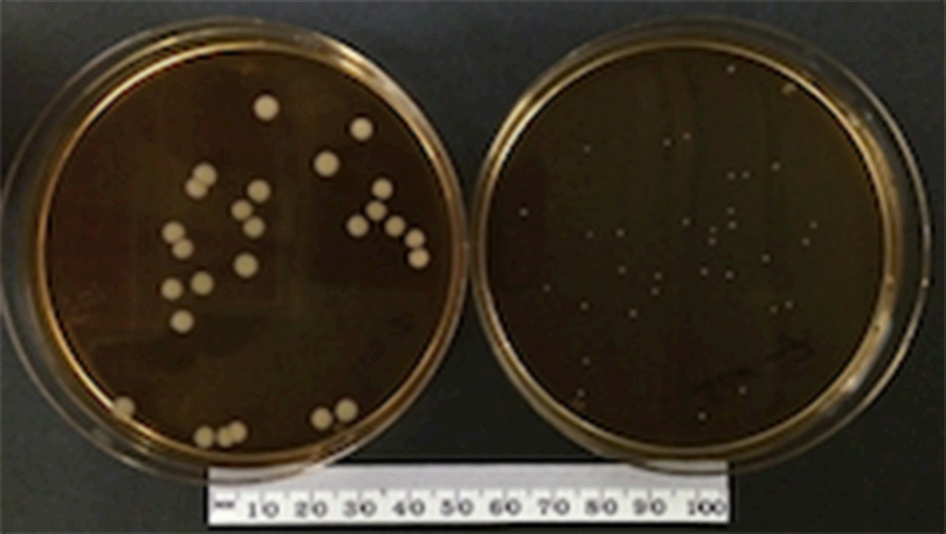
**WCH-SK2^WT^ and WCH-SK2^SCV^ colony**. Culture of *S. aureus* WCH-SK2^WT^ **(left)** and WCH-SK2^SCV^ **(right)** on brain heart infusion agar at 37°C for 24 h. WCH-SK2^WT^ = *Staphylococcus aureus* WCH-SK2 strain wild type, WCH-SK2^SCV^ = *Staphylococcus aureus* WCH-SK2 strain small colony variant.

*S. aureus* WCH-SK2 wild type (WCH-SK2^WT^) is a clinically isolated strain in blood culture from a patient with sepsis. This wild type parental strain was found to have increasing number of stable SCVs after prolonged culture under nutrient poor environment (Bui et al., 2015a). The frozen stocks of WCH-SK2^WT^ and WCH-SK2 Day50 (D50) were kindly donated by SK (University of Adelaide). A population of stable SCVs from WCH-SK2 D50 were isolated, termed WCH-SK2^SCV^, and used in the subsequent infection experiments. Both WCH-SK2^WT^ and WCH-SK2^SCV^ strains were cultured on brain heart infusion (BHI) agar at 37°C for 24 h (Figure 1). A single colony from each strain was then cultured in BHI broth at 37°C overnight with agitation.

### Eukaryotic cell culture

The human bronchial epithelial cell line, NuLi-1 (CRL-4011), was chosen for the following intracellular infection experiments and gene expression study as NuLi-1 cells have been shown to possess conserved TLR gene expression and innate immune response pathways when compared to primary human airway epithelial cells (Cooksley et al., 2015). The cells were purchased from ATCC and cultured in bronchial epithelial growth medium, BEGM (CC-3170, Lonza, Switzerland) supplemented with G-418 at 50 μg/ml. The cells were cultured in collagen (04902, Stemcell Technologies, Canada) coated T75 flasks. Passage 4 and 5 cells were used for all *S. aureus* infections.

### *S. aureus* intracellular infection optimization

**Figure 2:**
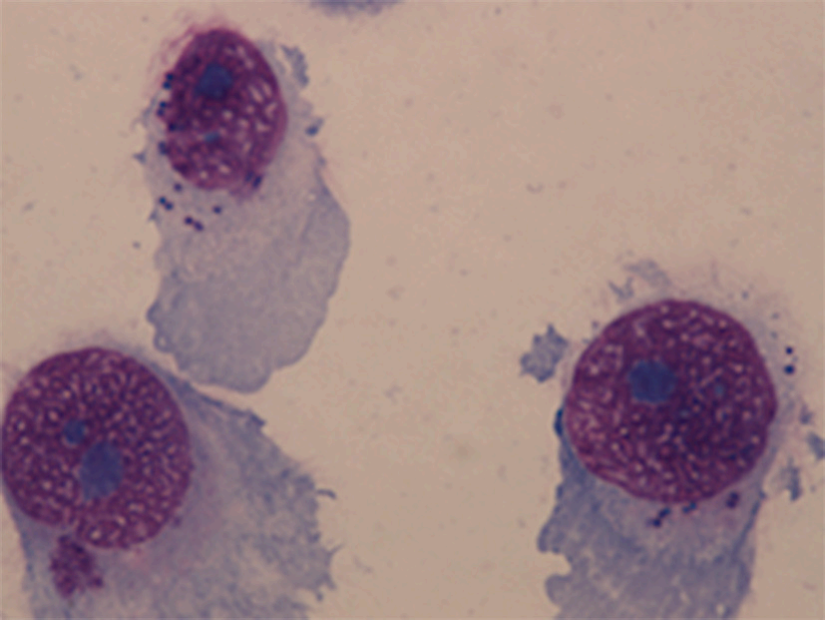
**NuLi-1 cells with intracellular *S. aureus***. NuLi-1 cells were incubated with *S. aureus* for 6 h, followed by lysostaphin treatment. The cells were fixed using methanol and stained by Giemsa stain. The cells were examined using a light microscope at 100X magnification with immersion oil. *S. aureus* was stained dark purple within the cytoplasm.

NuLi-1 cells were seeded onto collagen coated 8-chamber culture slides (FAL354108, Corning, USA) at 1.6 × 10^5^ cells/ml for 24 h prior to *S. aureus* infection. Overnight BHI broth culture of *S. aureus* WCH-SK2^WT^ and WCH-SK2^SCV^ were centrifuged at 3080 × g for 10 min at room temperature and pellets were resuspended in 0.9% saline and made up to McFarland Unit (MFU) 2, 3, and 4 standards. The bacterial solution was diluted 1:12.5 in BEGM (which was equivalent to MOI 50, 100, and 150 respectively) and incubated with NuLi-1 cells for 1, 2, 3, 4, 6, and 8 h at 37°C with 5% CO_2_ and 90% humidity. The medium containing bacteria was removed at the end of the incubation and the cells were washed twice using PBS buffer. Lysostaphin (L7386, Sigma-Aldrich, USA) was then added to the medium at 4 μg/ml for 30 min to lyse any remaining extracellular *S. aureus*. The cell culture continued for another 24 h with gentamycin (15750-060, Gibco Fisher Scientific International, USA) at 100 μg/ml. After 24 h the cells were fixed using methanol for 7 min. NuLi-1 cells were stained using Giemsa stain for 1 h and the slides were examined using a light microscope (Eclipse 90i, Nikon, Japan) at 100X magnification with immersion oil. Minimally 200 NuLi-1 cells were counted from each well to determine the intracellular infection rate (Figure 2). Lactate dehydrogenase (LDH) assays were performed using cell supernatants to determine cell viability as per the manufacturer's instruction (G1780, Promega, USA). Cell viability was calculated in reference to NuLi-1 cells at time 0 prior to any treatment. Three separate experiments in duplicate were performed.

### *S. aureus* infection for gene expression analysis

NuLi-1 cells were seeded into 12-well plates (PIMWS1250, EMD Millipore, USA) as per 8-chamber culture slides. There were two arms of treatment- extracellular vs. intracellular infections. For each arm the cells were treated with either no bacteria (negative controls), *S. aureus* WCH-SK2^WT^ (MOI 100) or *S. aureus* WCH-SK2^SCV^ (MOI 100). For extracellular infections, *S. aureus* was added to BEGM for the entire 24 h of incubation without lysostaphin or gentamycin treatment. The intracellular infection protocol was as per the intracellular optimization experiment. *S. aureus* solution in BEGM (MOI 100) was added to the cells for 6 h incubation followed by 30 min of lysostaphin treatment and 17.5 h of incubation with gentamycin added to BEGM. Four independent experiments with triplicates within each treatment were performed.

### RNA extraction and real-time polymerase chain reaction (PCR)

NuLi-1 cells were lysed at 24 h using 350 μl buffer RLT (79216, Qiagen, Germany) containing 3.5 μl 2-mercaptoethanol (M3148, Sigma-Aldrich, USA) per well. RNA extraction was performed using RNeasy Mini Kit (74106, Qiagen, Germany) as per the manufacturer's instructions. Thirty-nine genes (Supplementary Table 1) involved in innate and adaptive immune response as well as tissue remodeling were selected for gene expression analysis. Quantitect Reverse Transcription Kit (205313, Qiagen, Germany) was used for cDNA synthesis as per the manufacturer's instructions. The cDNA was pre-amplified with the 39-pooled Taqman gene expression assays for 16 cycles of 95°C for 15 s and 60°C for 4 min. The pre-amplified cDNA was diluted 1:5 in DNA Suspension Buffer (T0221, TEKnova Inc, CA, USA). Samples were prepared using 2.25 μl diluted cDNA, 2.5 μl 2X Master Mix and 0.25 μl 20X GE Sample Loading Reagent (85000746, Fluidigm Corporation, CA, USA). Gene assays were prepared by mixing 2.5 μl 20X Taqman Gene Expression Assays with 2.5 μl 2X Assay Loading Reagent (85000746, Fluidigm Corporation, CA, USA). Both samples and assays were loaded on to a 48.48 Dynamic Array chip (BMK-M-48.48, Fluidigm Corporation, CA, USA) and primed into the matrix using IFC Controller MX (Fluidigm Corporation, CA, USA). Real-time PCR was conducted using Biomark HD Platform (Fluidigm Corporation, CA, USA) and programmed as follows: 95°C for 1 min and 35 cycles of 96°C for 5 s and 60°C for 20 s. Data acquisition and analysis was performed using Fluidigm Real-Time PCR Analysis Software v4.1.2 (Fluidigm Corporation, CA, USA). Delta-cycle threshold (ΔCt) values were calculated in reference to the hypoxanthine phophoribosyltransferase (HPRT) 1 housekeeping gene. Delta-ΔCt (ΔΔCt) values were calculated in reference to negative control cells and all data was presented as fold change (2^−ΔΔCt^).

### Statistical analysis

Statistical analysis was performed using IBM SPSS Statistics, Version 23.0 (IBM Corp., Armonk, NY, USA). Kruskal–Wallis test was used to compare the intracellular infection of *S. aureus* of different MOI and incubation time as well as cell viability post *S. aureus* infection. A two-tailed *t*-test with permutation was used to analyse the relative gene expression fold changes for WCH-SK2^WT^ and WCH-SK2^SCV^ in comparison to negative control cells. Statistical significance was defined as *p* < 0.05.

## Results

### Comparison of intracellular infection rate of WCH-SK2^WT^ and WCH-SK2^SCV^

**Figure 3:**
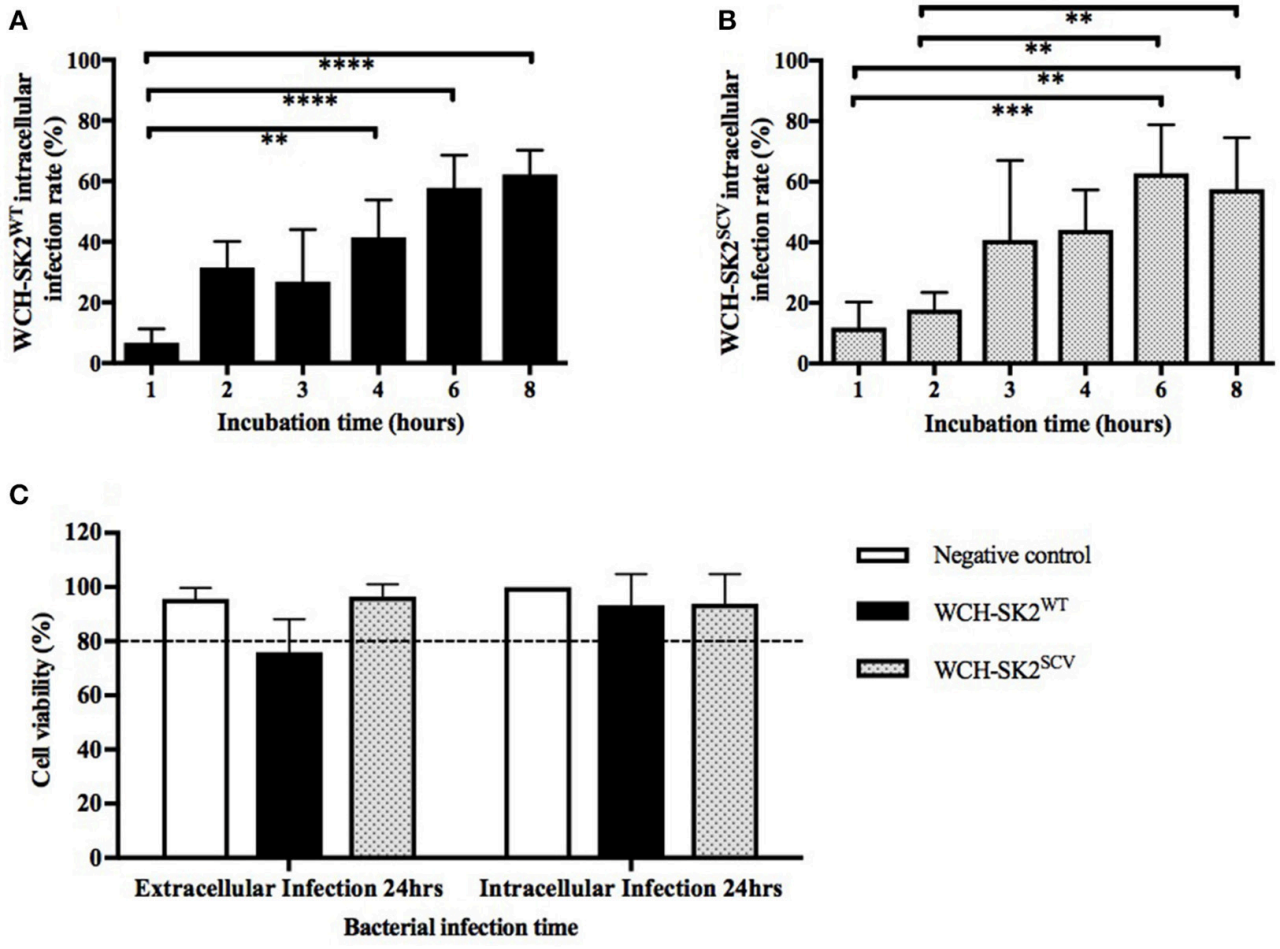
**WCH-SK2^WT^ and WCH-SK2^SCV^ intracellular infection rate and cell viability post infection**. Comparison of intracellular infection rate in NuLi-1 cells between *S. aureus* WCH-SK2^WT^ **(A)** and WCH-SK2^SCV^ **(B)** strains (MOI 100). NuLi-1 cells were incubated with either WCH-SK2^WT^ and WCH-SK2^SCV^ for a variable incubation time. The cells were treated with lysostaphin to remove any extracellular *S. aureus* and the incubation was continued with gentamycin. The intracellular infection rate was determined 24 h post lysostaphin treatment. The data shown is mean ± *SD* of three independent experiments measured. ^**^p ≤ 0.01, ^***^p ≤ 0.001, ^****^p ≤ 0.0001, Kruskal–Wallis test. NuLi-1 cell viability post extracellular and intracellular *S. aureus* infection was determined using an LDH assay **(C)**. For extracellular infections, the *S. aureus* was incubated with the cells for 24 h. For intracellular infections, the *S. aureus* was incubated with the cells for 6 h followed by lysostaphin treatment. The cells were incubated with gentamycin for another 17.5 h for a total of 24 h of incubation respectively. The data shown is mean ± *SD* of three independent experiments measured. Kruskal–Wallis test. LDH = Lactate dehydrogenase, WCH-SK2^WT^ = *Staphylococcus aureus* strain WCH-SK2 wild type. WCH-SK2^SCV^ = *Staphylococcus aureus* strain WCH-SK2 small colony variant.

The intracellular infection rate was found to increase with increasing bacterial incubation times with NuLi-1 cells for both WCH-SK2^WT^ and WCH-SK2^SCV^. As shown in Figures 3A,B, for both WCH-SK2^WT^ and WCH-SK2^SCV^ strains, the intracellular infection rate of NuLi-1 cells was less than 30% after 1 or 2 h incubation, and increased significantly after 4 h (41–44%, *p* ≤ 0.001) and 6 h (58–63%, *p* ≤ 0.001) incubation. Longer incubation times of up to 8 h did not further improve infection rates which remained at around 60%. There was no difference found in intracellular infection rate of NuLi-1 cells between WCH-SK2^WT^ and WCH-SK2^SCV^. Furthermore, when comparing different concentrations of bacteria, there was no difference found; using MOI 50, 100, and 150 at the same given incubation time. Overall, the time of bacterial incubation with NuLi-1 cells was found to be a more important factor to increase intracellular infection rate than bacterial concentration.

### Host cell viability during *S. aureus* infection

LDH cell toxicity assays showed the absence of significant toxicity for both WCH-SK2^WT^ and WCH-SK2^SCV^ extracellular and intracellular infection of NuLi-1 cells compared to control cells for 24 h (*p* > 0.05). The viability was greater than 75% for extracellular bacterial challenge and was greater than 90% for intracellular infection of NuLi-1 cells with WCH-SK2^WT^ and WCH-SK2^SCV^ for 24 h (Figure 3C).

### Differential gene expression profiles of infected cells

Gene expression was normalized to the housekeeping gene *HPRT1* and compared between negative controls and cells exposed to different bacterial challenges. A total of thirty-nine genes were tested. Seven of these genes, *CCL11, IL10, IL17A, IL25, interferon gamma (INFG), toll like receptor (TLR) 1*, and *TNF* were not expressed in any of the samples. Three genes, *IL33, lymphotoxin alpha (LTA)*, and *NLR family pyrin domain containing 3 (NLRP3)* were expressed at a very low levels in a few samples only and undetectable in the remaining samples.

### The influence of *S. aureus* WCH-SK2^WT^ and WCH-SK2^SCV^ intracellular infection

**Figure 4:**
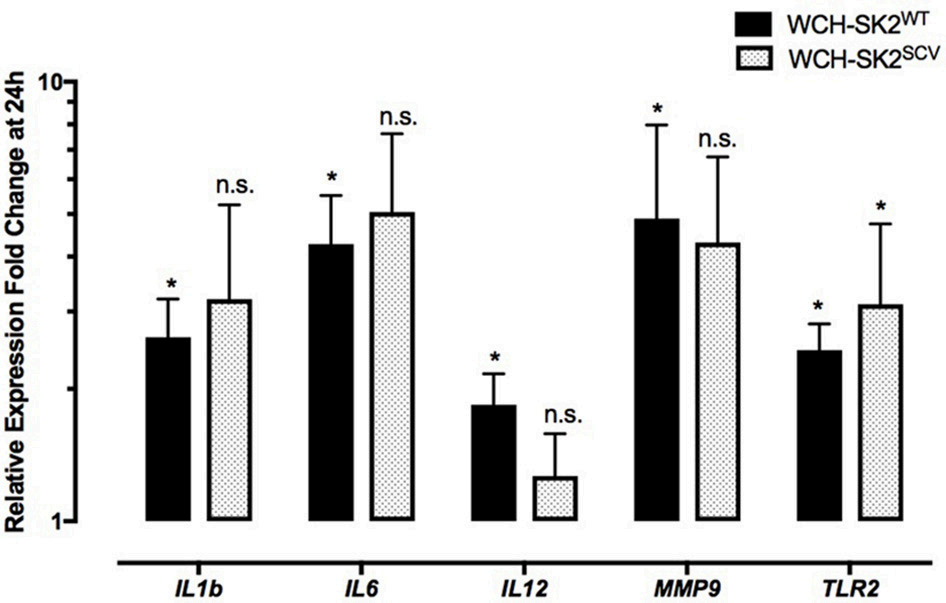
**mRNA gene expression changes in NuLi-1 cells at 24 h of WCH-SK2^WT^ and WCH-SK2^SCV^ intracellular infection**. The cells were incubated with *S. aureus* for 6 h (MOI 100), followed by lysostaphin treatment. The cells were incubated with gentamycin for another 17.5 h prior cell lysis and RNA extraction. The values represent the mean ± *SD* of four independent experiments measured. ^*^p ≤ 0.05, n.s. = non-significant, two tailed *t*-test with permutation between either WCH-SK2^WT^ or WCH-SK2^SCV^ vs. negative controls. WCH-SK2^WT^ = *Staphylococcus aureus* strain WCH-SK2 wild type. WCH-SK2^SCV^ = *Staphylococcus aureus* strain WCH-SK2 small colony variant.

Differential gene expression in WCH-SK2^WT^ intracellularly infected cells in comparison to negative control cells at 24 h is summarized in Supplementary Table 2. Up regulation was found for *IL1B* (fold change 2.62, *p* = 0.04), *IL6* (fold change 4.27, *p* = 0.04), *IL12* (fold change 1.84, *p* = 0.04), *MMP9* (fold change 4.88, *p* = 0.04) and *TLR2* (fold change 2.45, *p* = 0.04) in NuLi-1 cells infected with intracellular WCH-SK2^WT^ (Figure 4).

The same comparison was made between WCH-SK2^SCV^ intracellularly infected cells and negative controls cells at 24 h (Figure 4). Up regulation was only found for *TLR2* (fold change 3.12, *p* = 0.04). Overall, there was less widespread immune activation with intracellular WCH-SK2^SCV^ than WCH-SK2^WT^ invasion.

### WCH-SK2^WT^ and WCH-SK2^SCV^ extracellular infection

**Figure 5:**
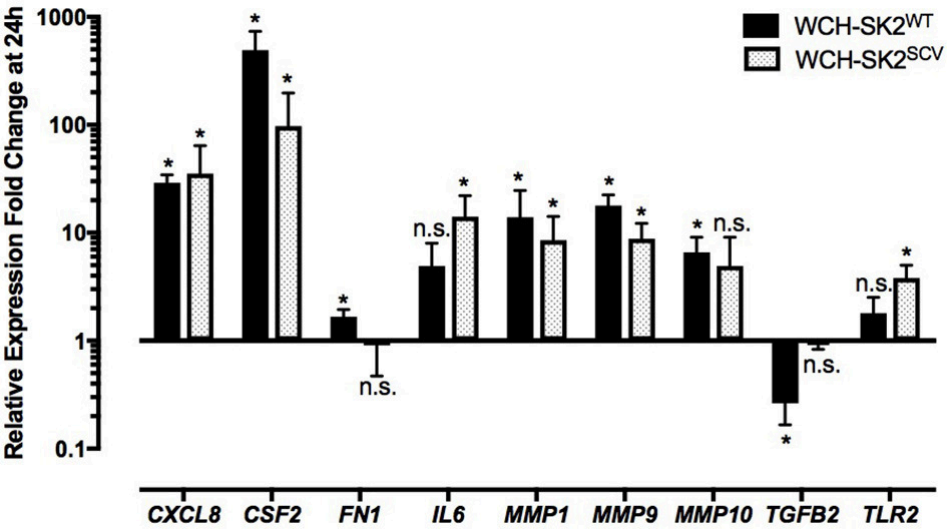
**mRNA gene expression changes in NuLi-1 cells at 24 h of WCH-SK2^WT^ and WCH-SK2^SCV^ extracellular infection**. The cells were incubated with *S. aureus* for 24 h (MOI 100), followed by cell lysis and RNA extraction. The values represent the mean ± *SD* of four independent experiments measured. ^*^p ≤ 0.05, n.s. = non-significant, 2 tailed *t*-test with permutation between either WCH-SK2^WT^ or WCH-SK2^SCV^ vs. negative controls. WCH-SK2^WT^ = *Staphylococcus aureus* strain WCH-SK2 wild type. WCH-SK2^SCV^ = *Staphylococcus aureus* strain WCH-SK2 small colony variant.

Up regulation of *CSF2* (fold change 492.1, *p* = 0.05), *CXCL8* (fold change 29.10, *p* = 0.05), *fibronectin (FN) 1* (fold change 1.67, *p* = 0.05), *MMP1* (fold change 13.90, *p* = 0.05*), MMP9* (fold change 17.89, *p* = 0.05) and *MMP10* (fold change 6.58, *p* = 0.05) was found in cells infected with WCH-SK2^WT^ extracellularly for 24 h in comparison to negative controls (Figure 5). On the other hand, *transforming growth factor beta (TGFB) 2* was down regulated (fold change 0.26, *p* = 0.05) in WCH-SK2^WT^ infected cells.

*CSF2* (fold change 97.01, *p* = 0.04), *CXCL8* (fold change 35.34, *p* = 0.04, *IL6* (fold change 14.12, *p* = 0.04), *MMP1* (fold change 8.53, *p* = 0.04), *MMP9* (fold change 8.81, *p* = 0.04) and *TLR2* (fold change 3.81, *p* = 0.04) were significantly up regulated in NuLi-1 cells infected with WCH-SK2^SCV^ extracellularly for 24 h (Figure 5). Details of all the changes in gene expression in cells with WCH-SK2^WT^ and WCH-SK2^SCV^ extracellular infection has been included in Supplementary Table 3.

### The influence on gene expression by extracellular vs. intracellular infection

**Figure 6:**
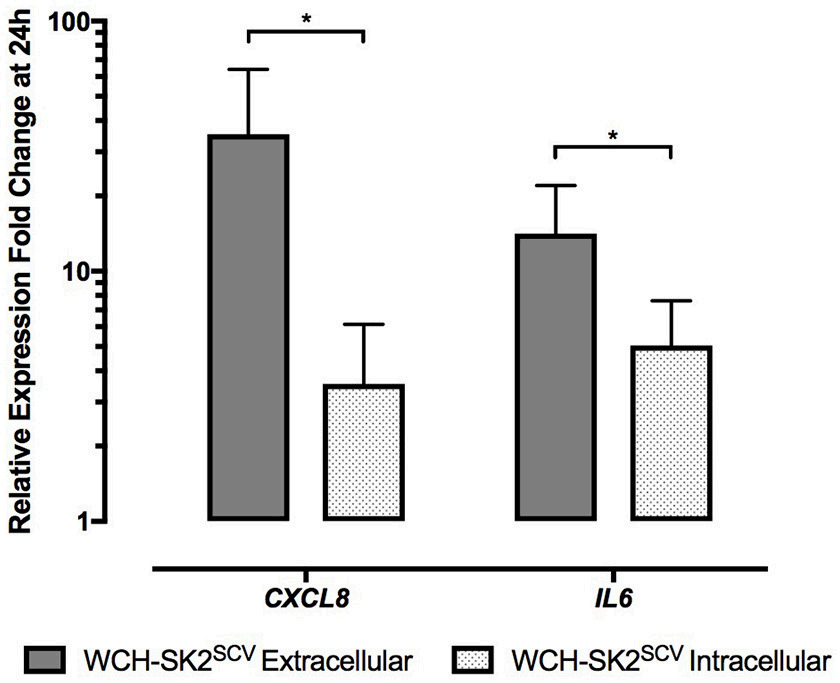
**mRNA gene expression changes in NuLi-1 cells at 24 h of WCH-SK2^SCV^ extracellular vs. intracellular infection**. The cells were incubated with *S. aureus* for either 24 h (extracellular infection) or 6 h (intracellular infection) followed by lysostaphin treatment and 17.5 h of incubation with gentamycin. The values represent the mean ± *SD* of four independent experiments measured. ^*^p ≤ 0.05, two tailed *t*-test with permutation between WCH-SK2^SCV^ extracellular and intracellular infections. WCH-SK2^SCV^ = *Staphylococcus aureus* strain WCH-SK2 small colony variant.

In WCH-SK2^SCV^ infected cells, *CXCL8* (fold change 35.34 vs. 3.55 for extracellular and intracellular infection respectively, *p* = 0.04), and *IL6* (fold change 14.12 vs. 5.05 for extracellular and intracellular infection respectively, *p* = 0.04) expression was significantly higher in extracellular than intracellular infected cells (Figure 6).

When comparing extracellular vs. intracellular infection of WCH-SK2^WT^, there was no significant difference found in gene expression for any of the genes tested.

## Discussion

This study was conducted to evaluate the differences between a clinically isolated *S. aureus* strain, WCH-SK2^WT^, and its laboratory derived SCV strain, WCH-SK2^SCV^, in terms of their ability to invade epithelial cells as well as the subsequent host cellular response they elicit. Extracellular *S. aureus* infection with both WCH-SK2^WT^ and WCH-SK2^SCV^ induced a similar up regulation of innate immune cytokines/chemokines (e.g., *CXCL8* and *IL1B*) as well as tissue remodeling genes (e.g., *MMP9*) within 24 h. With intracellular *S. aureus* infection, while the pattern of gene expression change was similar between both variants, WCH-SK2^WT^ induced a more widespread innate immune response than WCH-SK2^SCV^.

Adherence of *S. aureus* to host cells is the first vital step for host intracellular invasion. *S. aureus* fibronectin-binding proteins (FnBPs) are a group of the adhesins that have been most widely studied. It has been shown that binding of FnBPs to host fibronectin leads to activation of integrin mediated signaling pathways, reorganization of the actin cytoskeleton, and internalization of *S. aureus* (Agerer et al., 2005; Selbach and Backert, 2005; Schroder et al., 2006). *S. aureus* lacking FnBPs were found to have a significantly reduced internalization rate in bovine epithelial cells and in a mouse mastitis model (Brouillette et al., 2003a,b). ECM-binding protein homolog (Ebh) is a 1.1-megadalton *S. aureus* protein that binds to fibronectin, and was previously found to be significantly up regulated in WCH-SK2^SCV^ (log_2_ 5.3 fold) in comparison to WCH-SK2^WT^ (Bui et al., 2015a). Despite this up regulation and the notion that *S. aureus* isolated from within host cells are frequently SCVs (Tan et al., 2014) and can survive within the intracellular environment (Tuchscherr et al., 2010), no increase in intracellular infection rate of WCH-SK2^SCV^ compared to WCH-SK2^WT^ was found in our study. This finding is in line with other reports that failed to show an alteration in virulence of *ebh* mutant strains (Clarke et al., 2002) and indicates that the Ebh protein is not critical for intracellular invasion of *S. aureus*. Also, it has been shown that only some SCV strains have increased infection rates compared to their wild-type counterparts and that both the mammalian cell type and SCV strain can influence infection rate and survival of SCVs within the host (Tuchscherr et al., 2010).

TLRs are transmembrane proteins that recognize pathogen associated-molecular patterns (PAMPs) and play a crucial role in innate immune responses. TLR2 is the main receptor for *S. aureus* recognition (Zahringer et al., 2008). In our study, *TLR2* (but not *TLR6*) was found to be up regulated in both *S. aureus* WCH-SK2^WT^ and WCH-SK2^SCV^ infection (i.e., intracellular WCH-SK2^WT^ and WCH-SK2^SCV^, and extracellular WCH-SK2^SCV^ infection). TLR2 forms heterodimers with TLR1 or TLR6 to bind to either di-acylated (TLR2/TLR6 heterodimers) or tri-acylated lipopeptides (TLR2/TLR1 heterodimer) (Jin et al., 2007) and are involved in *S. aureus* peptidoglycan recognition in macrophages (Ozinsky et al., 2000; Nishiya and Defranco, 2004). A relative increase of TLR2 expression in airway epithelial cells has been shown to augment the innate immune response of these cells to further TLR2 agonists, as can be expected (Melkamu et al., 2009). Previous research has also shown that signaling through the TLR2/TLR6 heterodimer induced by *S. aureus* can induce IL10 production and apoptosis of antigen-presenting cells and is used by the bacteria to evade the immune response initiated by its own exotoxins (Takeda and Akira, 2005; Chau et al., 2009). Whether the increased *TLR2* expression in *S. aureus* infected airway epithelial cells translates to an altered sensitivity of these cells to specific microbial triggers and how this affects the immune response to itself and to different microbial organisms is unclear and warrants further investigation.

In our study, in comparison to intracellular infection, which induced a modest pro-inflammatory response as reported by others (Tuchscherr et al., 2011; Tan et al., 2014), extracellular infection induced manifest changes in gene expression of pro-immune factors (such as *CSF2, CXCL8*) and of tissue remodeling factors (such as *MMP1, MMP9*) as expected (Wang et al., 2010). The combined effect of these factors is hypothesized to enhance a Th1-type immune response facilitating neutrophil recruitment (Van Den Steen et al., 2000).

Intracellular infection with the *S. aureus* WCH-SK2^WT^ elicited a more consistent innate immune response in comparison to intracellular *S. aureus* WCH-SK2^SCV^ infection with an increased expression of *IL1B, IL6, IL12, MMP9*, and *TLR2* at 24 h. In contrast, intracellular WCH-SK2^SCV^ infection only induced up regulation of *TLR2* but not any significant cytokines expression after 24 h of infection. This data indicates that while the wild type *S. aureus* was able to invade NuLi-1 cells, it elicited a more widespread innate response which can lead to earlier host elimination. This finding is consistent with a study conducted by von Eiff et al. in which they showed that wild type *S. aureus* was able to invade host cells but did not survive as long as SCVs within the intracellular environment (Von Eiff et al., 2001).

*S. aureus* has been shown to induce the tissue remodeling factors MMP2, MMP9, and TIMP1 in mucosal explants from chronic rhinosinusitis (CRS) patients (Wang et al., 2010). Similarly, we also found an up regulation of tissue remodeling factors induced by extracellular infection with WCH-SK2^WT^ (*MMP1, MMP2*, and *MMP10*) and WCH-SK2^SCV^ (*MMP1* and *MMP9*) as well as intracellular infection with WCH-SK2^WT^ (*MMP9*). The exact mechanism of how *S. aureus* induces tissue remodeling is not fully understood. A study conducted by Pender and Macdonald has shown that *S. aureus* enterotoxin B was associated with up regulation of MMPs in fetal mesenchymal cells (Pender and Macdonald, 1998). WCH-SK2^WT^ and WCH-SK2^SCV^ are known to produce enterotoxin A, K, and L when cultured in liquid broth (unpublished data) (Bui et al., 2015a). Interestingly, in our experiments, *MMP9* up regulation was significantly higher in extracellular WCH-SK2^WT^ infection compared to intracellular WCH-SK2^WT^ infection and *MMP9* was not significantly induced by intracellular infection with the WCH-SK2^SCV^ variant. This finding could be explained by previous research that showed that some *S. aureus* strains can switch off or reduce their enterotoxin production upon host cell invasion and SCV formation (Tan et al., 2014). It is also known that TGFB can modify the expression, secretion, and activation of MMPs, which can reciprocally activate latent TGFB affecting the balance of ECM remodeling (Krstic and Santibanez, 2014). Our results, however, show a down regulation of *TGFB2* and up regulation of *MMP*s in NuLi-1 cells infected with extracellular WCH-SK2^WT^, potentially indicating a negative feedback loop. Further research is needed to determine the molecular entities and pathways activated that determine the increased expression of MMP9 by epithelial cells upon encounter with *S. aureus*.

Since we aimed to compare gene expression changes between different bacterial strains, and for reasons of feasibility and reproducibility, in our study, we used a normal bronchial epithelial cell line (NuLi-1 cells) rather than primary nasal epithelial cells (HNECs). Previous studies have indeed confirmed these cells to respond to different TLR agonists, confirming their capacity to respond to microbial stimulations (Cooksley et al., 2015). Some differences in the extent of their innate immune response to some TLR agonists when compared to HNECs have been observed however (Cooksley et al., 2015). These differences could be due to the fact that NuLi-1 cells have been transformed and are of bronchial origin, compared to HNECs, which are derived from the nasal mucosa. Further experiments using primary cell cultures should therefore be conducted to confirm the specific individual gene expression changes induced by different *S. aureus* strains.

## Conclusion

Our study demonstrated that the WCH-SK2^SCV^ strain did not have increased intracellular infection rate in comparison to its wild type parent strain, WCH-SK2^WT^. While both WCH-SK2^WT^ and WCH-SK2^SCV^ were able to invade and reside within NuLi-1 cells, the innate immune response to WCH-SK2^WT^ was more robust compared to the response elicited by WCH-SK2^SCV^. This provides further evidence supporting the notion that *S. aureus* SCVs are better equipped to evade immune detection and elimination by host cell.

## Author contributions

All authors in the manuscript have contributed significantly to from the design of the study to the completion of the manuscript. Study conception and design: JO, SV. Acquisition and analysis of data: JO, AD, CC. Interpretation of data: JO, AD, CC, AB, SK, AP, PW, SV. Drafting and critical revision: JO, AD, CC, AB, SK, AP, PW, SV. Final approval: JO, AD, CC, AB, SK, AP, PW, SV. Agreement to be accountable for all aspects of the work: JO, AD, CC, AB, SK, AP, PW, SV. No assistance in writing other than copy editing was used in the preparation of the manuscript.

## Funding

This work was supported by a Project Grant to PW by The Garnett Passe and Rodney Williams Memorial Foundation.

### Conflict of interest statement

The authors declare that the research was conducted in the absence of any commercial or financial relationships that could be construed as a potential conflict of interest.
