# Supplementary material for: Reduced Innate Immune Response to a Staphylococcus aureus Small Colony Variant Compared to Its Wild-Type Parent Strain
Source: Front Cell Infect Microbiol. 2016 Dec 26;6:187. doi: 10.3389/fcimb.2016.00187 (PMC5183720; doi:10.3389/fcimb.2016.00187)
Supplement: Supplementary file 1 [file Table1.DOCX]

**Supplementary Table 1. List of the Taqman Gene Assays used for gene expression analysis.**

| Gene | Description | Assay no. |
| --- | --- | --- |
| *CCL5* | C-C motif chemokine ligand 5 | Hs00174575_m1 |
| *CCL11* | C-C motif chemokine ligand 11 | Hs00237013_m1 |
| *CXCL8* | C-X-C motif ligand 8, (previous known as interleukin 8) | Hs00174103_m1 |
| *CSF2* | colony stimulating factor 2 | Hs00171266_m1 |
| *CSF3* | colony stimulating factor 3 | Hs99999083_m1 |
| *FN1* | fibronectin 1 | Hs00277509_m1 |
| *HPRT1* | hypoxanthine phosphoribosyltransferase 1 | Hs03929098_m1 |
| *ICAM1* | intercellular adhesion molecule 1 | Hs00164932_m1 |
| *IL1B* | interleukin 1 beta | Hs00174097_m1 |
| *IL6* | interleukin 6 | Hs00985639_m1 |
| *IL10* | interleukin 10 | Hs00961622_m1 |
| *IL12A* | interleukin 12 alpha | Hs00168405_m1 |
| *IL17A* | interleukin 17A | Hs00174383_m1 |
| *IL25* | interleukin 25 | Hs03044841_m1 |
| *IL33* | interleukin 33 | Hs00369211_m1 |
| *INFG* | interferon gamma | Hs00989291_m1 |
| *LTA* | lymphotoxin alpha | Hs99999086_m1 |
| *LTB* | lymphotoxin beta | Hs00242737_m1 |
| *LTBR* | lymphotoxin beta receptor | Hs00158922_m1 |
| *MMP1* | matrix metalloproteinase 1 | Hs00899658_m1 |
| *MMP2* | matrix metalloproteinase 2 | Hs01548727_m1 |
| *MMP9* | matrix metalloproteinase 9 | Hs00234579_m1 |
| *MMP10* | matrix metalloproteinase 10 | Hs00233987_m1 |
| *NFKB1* | nuclear factor kappa B subunit 1 | Hs00765730_m1 |
| *NFKBIA* | NFKB inhibitor alpha | Hs00153283_m1 |
| *NLRP3* | NLR family pyrin domain containing 3 | Hs00918082_m1 |
| *NOD2* | nucleotide binding oligomerization domain containing 2 | Hs00223394_m1 |
| *PYCARD* | PYD and CARD domain containing protein | Hs01547324_gH |
| *RIPK2* | receptor interacting serine/threonine protein kinase 2 | Hs01572686_m1 |
| *TGFA* | transforming growth factor alpha | Hs00608187_m1 |
| *TGFB1* | transforming growth factor beta 1 | Hs00998133_m1 |
| *TGFB2* | transforming growth factor beta 2 | Hs00234244_m1 |
| *TGFB3* | transforming growth factor beta 3 | Hs01086000_m1 |
| *TIMP1* | TIMP metallopeptidase inhibitor 1 | Hs00171558_m1 |
| *TLR1* | toll like receptor 1 | Hs00413978_m1 |
| *TLR2* | toll like receptor 2 | Hs01872448_s1 |
| *TLR6* | toll like receptor 6 | Hs01039989_s1 |
| *TNF* | tumor necrosis factor | Ha99999043_m1 |
| *TSLP* | thymic stromal lymphopoietin | Hs00263639_m1 |
