# Supplementary material for: Reduced Innate Immune Response to a Staphylococcus aureus Small Colony Variant Compared to Its Wild-Type Parent Strain
Source: Front Cell Infect Microbiol. 2016 Dec 26;6:187. doi: 10.3389/fcimb.2016.00187 (PMC5183720; doi:10.3389/fcimb.2016.00187)
Supplement: Supplementary file 2 [file Table2.docx]

**Supplementary Table 2. Changes in mRNA gene expression in NuLi-1 cells at 24 hours of both WCH-SK2^WT^ and WCH-SK2^SCV^ intracellular infection.**

|  | WCH-SK2^WT^ | | | | WCH-SK2^SCV^ | | | |
| --- | --- | --- | --- | --- | --- | --- | --- | --- |
|  | Relative Expression Fold Change* | Lower CI | Upper CI | *p*-value^ | Relative Expression Fold Change* | Lower CI | Upper CI | *p*-value^ |
| ***CCL5*** | 25.4 | -7.3 | 58.08 | 0.20 | 8.89 | -8.3 | 26.09 | 0.20 |
| ***CSF2*** | 3.85 | -0.31 | 8.01 | 0.11 | 12.19 | -17.41 | 41.79 | 0.11 |
| ***CSF3*** | 3.11 | 0.87 | 5.68 | 0.34 | 3.65 | -1.27 | 8.57 | 0.34 |
| ***CXCL8*** | 1.85 | 1.42 | 2.28 | 0.11 | 3.55 | -0.58 | 7.67 | 0.06 |
| ***FN1*** | 1.67 | 0.81 | 2.52 | 0.20 | 1.41 | -0.29 | 3.11 | 0.89 |
| ***ICAM1*** | 3.91 | -1.77 | 9.6 | 0.49 | 3.38 | -3.95 | 10.7 | 0.89 |
| ***IL1B*** | 2.62 | 1.7 | 3.55 | 0.04 | 3.20 | -0.06 | 6.46 | 0.11 |
| ***IL6*** | 4.27 | 2.3 | 6.24 | 0.04 | 5.05 | 0.97 | 9.13 | 0.06 |
| ***IL12*** | 1.84 | 1.33 | 2.36 | 0.04 | 1.27 | 0.77 | 1.77 | 0.34 |
| ***LTB*** | 4.93 | -3.78 | 13.64 | 0.63 | 10.38 | -27.24 | 48 | 0.40 |
| ***LTBR*** | 1.46 | 0.13 | 2.79 | 0.99 | 1.27 | -0.76 | 3.3 | 0.89 |
| ***MMP1*** | 1.46 | 0.81 | 2.11 | 0.11 | 2.11 | -0.21 | 4.44 | 0.11 |
| ***MMP2*** | 1.00 | -0.13 | 2.12 | 0.49 | 1.07 | -1.63 | 3.77 | 0.86 |
| ***MMP9*** | 4.88 | -0.05 | 9.8 | 0.04 | 4.31 | 0.43 | 8.19 | 0.07 |
| ***MMP10*** | 2.15 | 0.22 | 4.08 | 0.20 | 2.66 | 0.24 | 5.08 | 0.11 |
| ***NFKB1*** | 1.16 | 0.76 | 1.56 | 0.34 | 1.23 | 0.51 | 1.94 | 0.69 |
| ***NFKBIA*** | 2.41 | 0.39 | 4.41 | 0.20 | 2.68 | -0.87 | 6.23 | 0.34 |
| ***NOD2*** | 4.69 | -0.8 | 10.18 | 0.11 | 6.52 | -2.28 | 15.32 | 0.11 |
| ***PYCARD*** | 1.21 | -0.05 | 2.47 | 0.99 | 1.33 | -0.28 | 3.95 | 0.89 |
| ***RIPK2*** | 1.21 | 0.65 | 1.76 | 0.49 | 1.33 | 0.73 | 0.92 | 0.49 |
| ***TGFA*** | 1.67 | 0.4 | 2.95 | 0.49 | 1.22 | -0.16 | 2.61 | 0.99 |
| ***TGFB1*** | 1.22 | 0.33 | 2.11 | 0.69 | 1.14 | -0.2 | 2.48 | 0.94 |
| ***TGFB2*** | 0.77 | 0.01 | 1.52 | 0.34 | 1.34 | -0.18 | 2.86 | 0.99 |
| ***TGFB3*** | 1.36 | -0.55 | 3.27 | 0.63 | 0.86 | 0.32 | 1.4 | 0.69 |
| ***TIMP1*** | 1.10 | 0.99 | 1.22 | 0.69 | 1.00 | 0.29 | 1.71 | 0.67 |
| ***TLR2*** | 2.45 | 1.88 | 3.03 | 0.04 | 3.12 | 0.53 | 5.71 | 0.04 |
| ***TLR6*** | 0.83 | 0.52 | 1.13 | 0.20 | 0.73 | 0.49 | 0.97 | 0.34 |
| ***TSLP*** | 1.11 | 0.64 | 1.59 | 0.34 | 1.08 | 0.59 | 1.56 | 0.49 |

| Significant up regulation | No change in expression |
| --- | --- |

* Fold change calculated as 2^-ΔΔCt^ in reference to respective negative controls

^ *p*-value obtained using independent 2-tailed *t*-test with permutation
