# Supplementary material for: Reduced Innate Immune Response to a Staphylococcus aureus Small Colony Variant Compared to Its Wild-Type Parent Strain
Source: Front Cell Infect Microbiol. 2016 Dec 26;6:187. doi: 10.3389/fcimb.2016.00187 (PMC5183720; doi:10.3389/fcimb.2016.00187)
Supplement: Supplementary file 3 [file Table3.docx]

**Supplementary Table 3. Changes in mRNA gene expression in NuLi-1 cells at 24 hours of both WCH-SK2^WT^ and WCH-SK2^SCV^ extracellular infection.**

|  | WCH-SK2^WT^ | | | | WCH-SK2^SCV^ | | | |
| --- | --- | --- | --- | --- | --- | --- | --- | --- |
|  | Relative Expression Fold Change* | Lower CI | Upper CI | *p*-value^ | Relative Expression Fold Change* | Lower CI | Upper CI | *p*-value^ |
| ***CCL5*** | 0.90 | -0.70 | 2.40 | 0.47 | 5.90 | -1.56 | 13.36 | 0.2 |
| ***CSF2*** | 492.10 | -105.50 | 1089.70 | 0.05 | 97.01 | -63.09 | 257.11 | 0.04 |
| ***CSF3*** | 0.70 | 0.30 | 1.20 | 0.52 | 19.40 | -8.88 | 47.62 | 0.34 |
| ***CXCL8*** | 29.10 | 16.20 | 42.00 | 0.05 | 35.34 | -10.54 | 81.23 | 0.04 |
| ***FN1*** | 1.67 | 1.00 | 2.30 | 0.05 | 0.90 | 0.21 | 1.60 | 0.69 |
| ***ICAM1*** | 4.11 | -7.30 | 15.50 | 0.64 | 9.70 | -2.35 | 21.77 | 0.2 |
| ***IL1B*** | 3.50 | -4.60 | 11.50 | 0.28 | 4.08 | 1.03 | 7.13 | 0.06 |
| ***IL6*** | 4.90 | -2.70 | 12.50 | 0.12 | 14.12 | 1.53 | 26.70 | 0.04 |
| ***IL12*** | 0.80 | -0.70 | 2.30 | 0.56 | 1.23 | 0.04 | 2.43 | 0.89 |
| ***LTB*** | 8.10 | -13.40 | 29.50 | 0.10 | 19.90 | -10.93 | 50.70 | 0.11 |
| ***LTBR*** | 1.00 | -0.20 | 2.20 | 0.39 | 1.40 | -0.15 | 2.91 | 0.68 |
| ***MMP1*** | 13.90 | -12.70 | 40.60 | 0.05 | 8.53 | -0.40 | 17.46 | 0.04 |
| ***MMP2*** | 0.60 | 0.00 | 1.30 | 0.20 | 1.14 | -0.58 | 2.44 | 0.47 |
| ***MMP9*** | 17.89 | 6.60 | 29.10 | 0.05 | 8.81 | 3.37 | 14.24 | 0.04 |
| ***MMP10*** | 6.58 | 0.34 | 12.82 | 0.05 | 17.69 | -23.27 | 58.66 | 0.20 |
| ***NFKB1*** | 0.62 | 0.50 | 0.70 | 0.16 | 1.10 | 1.06 | 1.14 | 0.90 |
| ***NFKBIA*** | 2.50 | -0.50 | 5.50 | 0.25 | 2.33 | -0.07 | 4.72 | 0.34 |
| ***NOD2*** | 1.20 | -1.00 | 3.50 | 0.83 | 2.60 | -0.28 | 5.18 | 0.68 |
| ***PYCARD*** | 0.60 | -1.00 | 2.20 | 0.29 | 1.30 | -0.22 | 2.80 | 0.99 |
| ***RIPK2*** | 0.80 | 0.40 | 1.10 | 0.36 | 1.50 | 0.94 | 2.08 | 0.34 |
| ***TGFA*** | 2.40 | -2.10 | 6.90 | 0.35 | 1.63 | 0.10 | 3.17 | 0.68 |
| ***TGFB1*** | 1.90 | 0.30 | 3.60 | 0.21 | 1.91 | 0.11 | 3.72 | 0.34 |
| ***TGFB2*** | 0.26 | 0.02 | 0.50 | 0.05 | 0.91 | 0.78 | 1.05 | 0.34 |
| ***TGFB3*** | 0.30 | -2.80 | 3.50 | 0.11 | 1.79 | -0.81 | 4.39 | 0.68 |
| ***TIMP1*** | 2.07 | 9.30 | 3.90 | 0.09 | 1.98 | 0.79 | 3.18 | 0.06 |
| ***TLR2*** | 1.80 | -0.01 | 3.60 | 0.21 | 3.81 | 1.91 | 5.71 | 0.04 |
| ***TLR6*** | 0.80 | -0.50 | 2.00 | 0.60 | 0.81 | -0.06 | 1.67 | 0.89 |
| ***TSLP*** | 2.90 | -5.10 | 11.00 | 0.58 | 1.50 | -0.10 | 3.04 | 0.69 |

| Significant up regulation | No change in expression | Significant down regulation |
| --- | --- | --- |

* Fold change calculated as 2^-ΔΔCt^ in reference to respective negative controls

^ *p*-value obtained using independent 2-tailed *t*-test
